# Dietary mineral intakes predict Coronavirus-disease 2019 (COVID-19) incidence and hospitalization in older adults

**DOI:** 10.1186/s40795-024-00821-5

**Published:** 2024-03-04

**Authors:** Najmeh Seifi, Hossein Bahari, Somayeh Ghiasi Hafezi, Farzaneh Ghotbani, AhmadReza Afzalinia, Gordon A. Ferns, Ehsan Mosa Farkhani, Majid Ghayour-mobarhan

**Affiliations:** 1 International UNESCO Center for Health-Related Basic Sciences and Human Nutrition, Mashhad University of Medical Sciences, Mashhad, Iran; 2 Department of Nutrition, Medical School, Mashhad University of Medical Sciences, Mashhad, Iran; 3 Transplant Research Center, Clinical Research Institute, Mashhad University of Medical Sciences, Mashhad, Iran; 4 Student Research committee, Mashhad University of Medical Sciences, Mashhad, Iran; 5 Department of Biology, Mashhad Branch, Islamic Azad University, Mashhad, Iran; 6 Brighton & Sussex Medical School, Division of Medical Education, Falmer, Brighton, Sussex UK; 7 Deputy of Health, Mashhad University of Medical Sciences, Mashhad, Iran; 8 Metabolic Syndrome Research Center, Mashhad University of Medical Sciences, Mashhad, Iran

**Keywords:** COVID-19, Diet, Nutrition, Mineral, Dynamical system

## Abstract

**Background:**

The aim of this study was to determine the association between dietary mineral intake and Coronavirus-disease 2019 (COVID-19) infection and its associated hospitalization.

**Methods:**

This cohort study utilized the MASHAD study population, which comprised individuals aged 35–65. Upon recruitment in 2007, dietary intake was documented using a validated 65-item food frequency questionnaire (FFQ). Data on COVID-19 PCR test results was collected from all relevant medical centers in Mashhad between February 2020 and June 2022. The regression model included dietary minerals and employed the backward variable selection method, along with advanced data analysis techniques.

**Results:**

The final analysis involved 1957 participants, including 193 COVID-19-positive patients. The mean age was 49.71 and 50.28 years in the COVID-19-positive and negative groups, respectively (*p* = 0.12). Dietary intakes of magnesium, iron, and potassium were notably lower in COVID-19-positive patients (*P* < 0.05). Following adjustments for age and sex, dietary iron remained significantly associated with COVID-19 incidence (OR = 0.94, 95% CI: 0.90–0.98). Furthermore, a statistically significant relationship was observed between dietary zinc and hospitalization due to COVID-19 (OR = 0.69, 95% CI: 0.51–0.93). In dynamical system models, intakes of calcium, zinc, and iron below the cut-offs of 1138, 9.7, and 8.17 mg/day, respectively, were linked to an increased risk of COVID-19 incidence.

**Conclusion:**

Higher dietary iron and zinc intake are associated with decreased risk of COVID-19 infection and hospitalization, respectively.

## Introduction

In 2020, the flare-up of Coronavirus-disease 2019 (COVID-19), attributed to the novel acute respiratory syndrome coronavirus-2 (SARS-CoV-2) virus, resulted in more than 633 million cases worldwide and 6 million deaths up until November 2022 [1]. Acute respiratory diseases are the leading cause of morbidity worldwide, with children and the elderly at increased risk, especially in low and middle-income countries [2, 3]. According to the latest statistics available on the Iranian population, as of June 22, 2022, 7.5 million people have been infected with COVID-19 (positive PCR test) and more than 140,000 deaths have been reported [4].

Individuals with an impaired immune system are at greater risk of acquiring infectious diseases, including COVID-19. Therefore, boosting the body's immune response is important in protecting against COVID-19. It is well-established that nutrition plays an important role in supporting the immune system. Based on both mechanistic and clinical literature, some vitamins, trace elements, including zinc, copper, iron, selenium, and magnesium, and fatty acids are essential for optimal function of the immune system at the level of physical barriers, innate and adaptive immunity, and the microbiome [5, 6]. Deficiencies in these essential micronutrients could increase the susceptibility to infectious diseases. However, nutrition does not impact all infectious diseases equally. In a study of COVID-19 patients, most patients were zinc deficient and had more complications than zinc-sufficient patients, including more acute respiratory distress syndrome, longer hospital stays, and increased mortality [7].

COVID-19 infection, due to its complex dynamic nature, cannot be adequately described by common linear analysis methods [8]. Nonlinear mathematical approaches, involving interactions, feedback loops, or non-proportional relationships between variables, have been effectively used for the analysis, understanding, and predictions of the dynamics of this pandemic [9, 10]. As among non-linear tools, dynamic system models provide valuable insights into the behavior and evolution of complex systems, enabling researchers and practitioners to make informed decisions, predict outcomes, and design interventions or control strategies over time [11], we operated this advanced data analysis method to predict the relationship between dietary mineral intake and COVID-19 incidence and hospitalization. To our knowledge, this is the first study in this area.

## Methods

### Study design

**Fig. 1:**
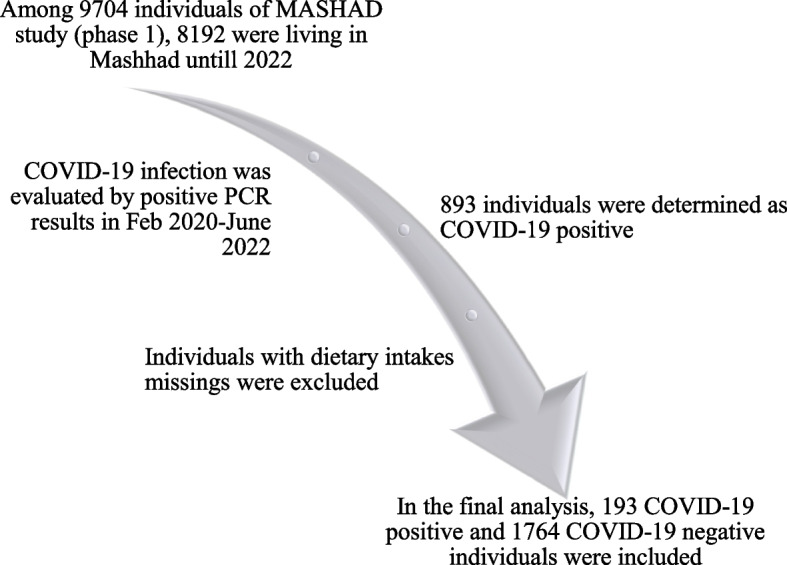
Flow diagram of study design

The population of this cohort study was derived from Mashhad stroke and heart atherosclerotic disorder (MASHAD) study [12]. Subjects who entered the first phase of MASHAD study (*n* = 9704, 2007–2010), who were also common in the second phase (2017–2020) and still living in Mashhad were included. All participants provided their written and informed consent. Our participants were apparently healthy and free of CVD, active cancers, chronic infections, or autoimmune disorders. We also excluded pregnant or lactating women. As previously described [13], to determine the COVID-19 infection, we collected the data of all PCR-positive patients from all related medical centers in Mashhad city during February 2020 and June 2022 (*n* = 405,398) and merged with MASHAD study data. Patients at baseline were 35–65 years old. After removing records with any missing dietary intake data, the remaining sample size was 1957, with 193 individuals testing positive for COVID-19 (Fig. 1). Hospitalized patients were identified using data from the hospital information system (HIS). The study protocol received approval from the Human Research Ethics Committee of Mashhad University of Medical Sciences (MUMS).

### Dietary intake assessment

Dietary intake was assessed using a validated 65-item food frequency questionnaire (FFQ) in the first phase of MASHAD study [14]. An experienced researcher administered the questionnaire, and data was collected on demographic characteristics. Diet plan software (version 6) was used to analyze the mineral intake [15]. Mineral intakes were energy-adjusted, using residual method [16].

### Statistical analysis

Data analysis was carried out by SPSS 26 (SPSS Inc., IL, USA). We utilized the Kolmogorov–Smirnov test to assess the normality of our variables. Normally distributed variables were presented as mean ± standard deviation, while non-normally distributed variables were described using median and interquartile range (IQR). We used multiple logistic regression with a backward variable selection method to determine the association of mineral intakes with COVID-19 infection and hospitalization. All analysis was performed in IBM SPSS statistics 26. Minerals including calcium, magnesium, phosphorus, iron, zinc, iodine, potassium, manganese, selenium, chloride, sodium, and copper were included in the model. Further, the effect of age and sex was adjusted. *P*-value < 0.05 was considered statistically significant.

Advanced data analysis methods were also applied to predict the relationship between dietary mineral intake and Coronavirus-disease COVID-19. First, the best cut-off values of mineral intakes, in regard to COVID-19 infection, were determined by MedCalc software. The best cut-off value is the point where the sensitivity + specificity – 1 is the maximum.

**Fig. 2:**
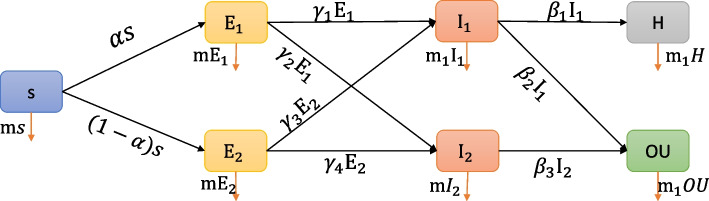
The modified SEIR (Susceptible-Exposed-Infected-Removed) model to predict COVID-19 infection and outcome. S: susceptible, E1: patients consuming minerals above the cut-off, E2: patients consuming minerals below the cut-off, I1: patients with positive PCR results for COVID-19, I2: patients with negative PCR results for COVID-19, H: hospitalized patients, OU: patients who were managed in outpatient setting, α: Rate of transmission from S to E1, 1-α: Rate of transmission from S to E2, m: rate of death in susceptible individuals,${\gamma }_{1}$:Rate of transmission from E1 to I1,${\gamma }_{2}:$ Rate of transmission from E1 to I2,${\gamma }_{3}$:Rate of transmission from E2 to I1, ${\gamma }_{4}:$ Rate of transmission from E2 to I2 $, {\beta }_{1}:$ Rate of transmission from I1 to H, ${\beta }_{2:}$ Rate of transmission from I1 to OU, ${\beta }_{3}:$ Rate of transmission from I2 to OU

The modified SEIR (Susceptible-Exposed-Infected-Removed) differential equation machine (Fig. 2) was utilized to forecast the impact of mineral intake on COVID-19 dynamics. In this particular model, all individuals were considered susceptible to contracting COVID-19 (S). Participants who consumed the mineral above the specified threshold are denoted as E1, while the group that consumed below the mentioned threshold is denoted as E2. I1 represents the group of individuals who tested positive for COVID-19 through PCR testing, while I2 refers to those who were not infected. When considering all participants within the modified equation model, this distinction is taken into account. In the aforementioned model, hospitalization due to COVID-19 (H) or outpatient management (OU) was also considered. To solve the differential equation described in the model, the numerical methods of ODe45 were employed within the Matlab software.

## Results

Baseline characteristics are presented in Tables 1 and 2. The median age of covid-19-positive and covid-19-negative subjects was 59 years. Around 49% of CPVID-19-positive and 46% of COVID-19-negative patients were female. As presented in Table 1, dietary intakes of magnesium, iron and potassium were significantly lower in Covid-19 patients compared to the COVID-19-negative group (*P* < 0.05). There was no significant difference in other variables.

**Table 1:** Baseline characteristics and mineral intakes in COVID-19 and non-COVID-19 patients

	**COVID-19-positive (*N* = 193)**	**COVID-19-negative *(N* = 1764)**	***p*-value**
Age, year	59(56,62)^a^	59(57,63)	0.12
Sex			
Female, n(%)	95 (49.2%)	819 (46.4%)	0.46
Calcium, mg/day	848.95 ± 323.91^b^	891.7 ± 356.26	0.14
Magnesium, mg/day	229.87 (186.09,277.35)	240.14 (200.96,294.49)	0.016*
Phosphorus, mg/day	1284.7 ± 354.35	1345 ± 349.40	0.05
Iron, mg/day	9.087 (6.712,12.268)	9.930 (7.563,13.477)	0.008*
Zinc, mg/day	8.859 (7.294,10.644)	9.033 (7.5244,10.957)	0.54
Iodine, μg/day	104.71 (55.95,160.47)	123.38 (52.69,168.64)	0.70
Potassium, mg/day	2653.5 (2160.4,3196.7)	2802.8 (2298.4,3415.7)	0.026*
Manganese, mg/day	3.750 (3.130, 4.516)	3.831 (3.143,4.643)	0.34
Selenium, μg/day	31.06 (22.81,41.73)	31.39 (23.30,43.66)	0.88
Chloride, mg/day	2874 (1536,4099)	2918 (1563,4099)	0.99
Sodium, mg/day	2390 (1412,3059)	2244 (1504,3029)	0.86
Copper, mg/day	1.82 (1.60,2.02)	1.81 (1.59,2.06)	0.89

^*^Significantly different

^a^Median (1st, 3st)

^b^Mean ± SD

**Table 2:** Baseline characteristics and mineral intakes in COVID-19 hospitalized and non-hospitalized patients

	**Hospitalization positive *(N* = 17)**	**Hospitalization negative *(N* = 176)**	***p*-value**
Age, year	61(56,63)^a^	58(56,62)	0.50
Sex			
Female, n (%)	6 (31.1%)	89 (46.1%)	0.22
Calcium, mg/day	878.9 (513.2, 1110.2)	852.03 (624.02,1022.8)	0.99
Magnesium, mg/day	204.42 ± 87.401	242.64 ± 98.90	0.21
Phosphorus, mg/day	1292.9 ± 436.67^b^	1334.3 ± 348.02	0.42
Iron, mg/day	7.827 ± 3.31	9.206 ± 4.63	0.09
Zinc, mg/day	7.84 (6.26,9.04)	9.37(7.33,10.84)	0.04
Iodine, μg/day	131.92 ± 78.32	113.90 ± 82.88	0.50
Potassium, mg/day	2380.7 ± 818.65	2821.3 ± 1127.97	0.10
Manganese, mg/day	3.41 ± 0.99	4.01 ± 1.81	0.09
Selenium, μg/day	31.40 (17.45,40.13)	31.06 (23.01,41.74)	0.62
Chloride, mg/day	2707 (2041.4,4373.7)	2874(1397,4068)	0.53
Sodium, mg/day	2227 (1776,2707)	2444(1376,3077)	0.94
Copper, mg/day	1.615 (1.44,1.92)	1.78 (1.57,2.02)	0.29

^a^Median(1st, 3st)

^b^Mean ± SD

The median age of hospitalized and non-hospitalized subjects was 61 and 58 years, respectively. Around 31% of hospitalized and 46% of non-hospitalized patients were female. As shown in Table 2, there was no significant difference in mineral intake of patients with and without hospitalization except for zinc, which was higher in subjects without hospitalization (*P* < 0.05).

In Table 3, a significant association was observed between the dietary intake of iron and phosphorus and the occurrence of COVID-19 (*P* < 0.05). Following adjustments for age and sex, iron continued to exhibit a significant association with COVID-19 incidence. Specifically, each unit increase in iron intake was linked to approximately a 6% reduction in the relative risk of COVID-19 infection.

**Table 3:** Relative risk and 95% confidence intervals for the association of mineral intakes and COVID-19 infection

Variables	Crude	*P*-value	Adjusted ^a^	*P*-value
	RR^b^ (95% CI^c^)		RR (95% CI)	
Zinc	0.9816(0.9229, 1.0440)	0.55	1.0512(0.9781,1.1298)	0.17
Iron	0.9444(0.9078,0.9825)	0.00	0.9414(0.9006,0.9840)	0.00
Phosphorus	0.9995(0.9990,1.0000)	0.04	0.9995(0.9989,1.0001)	0.10

^a^ Adjusted for age and sex

^b^ Relative Risk (RR)

^c^ Confident Interval (CI)

Prior to adjusting for age and sex, Table 4 demonstrates a significant association between dietary zinc intake and hospitalization (*P* < 0.05). This association retained its significance even after adjusting for age and sex (*P* < 0.05). Furthermore, each unit increase in dietary intake of zinc was linked to a 31% reduction in the relative risk of hospitalization due to COVID-19.

**Table 4:** Relative risk and 95% confidence intervals for the association of mineral intakes and COVID-19 hospitalization

Variables	Crude	*P*-value	Adjusted ^a^	*P*-value
	RR^b^ (95% CI^c^)		RR (95% CI)	
Calcium	1.000(0.9980,1.0020)	0.98	1.000(0.9969,1.0030)	0.98
Zinc	0.7343(0.5697,0.9465)	0.01	0.6941(0.5151, 0.9353)	0.01
Iodine	1.0023(0.9955, 1.0092)	0.50	1.0055(0.9950,1.0161)	0.30

^a^ Adjusted for age and sex

^b^ Relative risk (RR)

^c^ Confident Interval (CI)

### Dynamical system

The best cut-offs for mineral intake are presented in Table 5. The best cut-off for daily consumption of calcium, iodine, iron, phosphorus, and zinc were 1138.16 mg, 52.19 mg, 8.17 mg, 1439.35 mg, and 9.7 mg, respectively.

**Table 5:** Best cut-off points for daily mineral consumption

Variable	Best cutoff points	Sensitivity (%)	Specificity (%)	AUC (95% CI)
Calcium (mg)	1138.16	84.30	18.66	0.501(0.478, 0.515)
Iodine (μg)	52.19	30.45	73.56	0.517(0.503,0.531)
Iron (mg)	8.17	70.39	32.62	0.509(0.495,0.522)
Phosphorus(mg)	1439.35	70.37	32.94	0.504(0.490,0.518)
Zinc (mg)	9.70	44.88	62.38	0.536(0.522,0.550)

*AUC* area under the curve

Figure 2 represents the modified SEIR differential equation machine. If we consider all the participants in the modified equation model, we have the following equation:

$$\mathrm{N }\left({\text{t}}\right) =\mathrm{S }\left({\text{t}}\right) +{\text{E}}1 \left({\text{t}}\right) +{\text{E}}2 \left({\text{t}}\right) +{\text{I}}1 \left({\text{t}}\right) +{\text{I}}2 \left({\text{t}}\right) +\mathrm{H }\left({\text{t}}\right) +\mathrm{Ou }\left({\text{t}}\right)$$

Considering the mentioned model, the below differential equation was solved using the numerical methods of OD45 in the Matlab software.

1 $$\left\{\begin{array}{c}\dot{S}=\left(1+ {\text{m}}\right)s+L\\ \dot{{{\text{E}}}_{1}}=-\left(1+{\text{m}}\right){{\text{E}}}_{1}+\alpha s\\ \begin{array}{c}\dot{{{\text{E}}}_{2}}=-\left({\eta }_{3}+{\text{m}}\right){{\text{E}}}_{2}+(1-\alpha )s\\ \dot{{{\text{I}}}_{1}}={\gamma }_{1}{{\text{E}}}_{1}+{\gamma }_{3}{{\text{E}}}_{2}-\left({{\beta }_{1}+\beta }_{2}+{{\text{m}}}_{1}\right){{\text{I}}}_{1}\\ \begin{array}{c}\dot{{{\text{I}}}_{2}}={\gamma }_{2}{{\text{E}}}_{1}+{\gamma }_{4}{{\text{E}}}_{2}-m{{\text{I}}}_{2}-{\beta }_{3}{{\text{I}}}_{2}\\ \dot{H}={\beta }_{1}{{\text{I}}}_{1}-{{\text{m}}}_{1}H\\ \begin{array}{c}\dot{{\text{OU}}}={\beta }_{2}{{\text{I}}}_{2}-{{\text{m}}}_{1}OU+ {\beta }_{3}{{\text{I}}}_{2}\\ \dot{D}={\text{m}}s+{{\text{mE}}}_{1}+{{\text{mE}}}_{2}+{{\text{m}}}_{1}{{\text{I}}}_{1}+{{\text{m}}}_{1}{\text{H}}+{\text{m}}{I}_{2}+{{\text{m}}}_{1}{\text{OU}}\end{array}\end{array}\end{array}\end{array}\right.$$

**Fig. 3:**
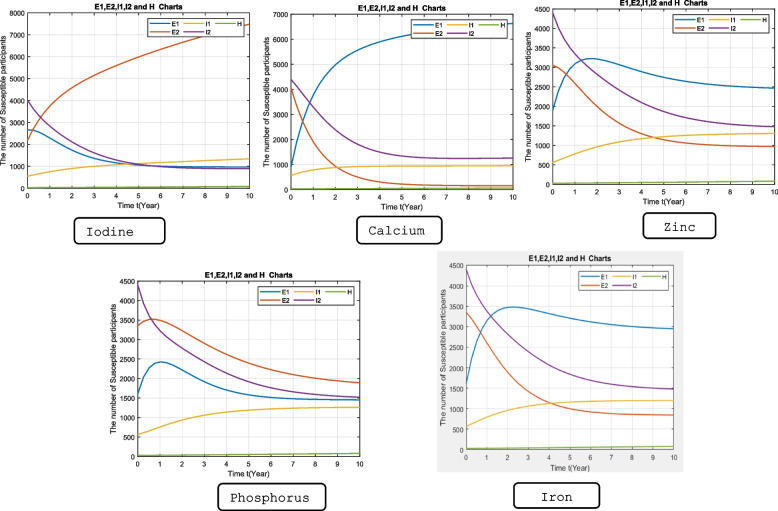
The model parameters for each mineral. E1: patients consuming minerals above the cut-off, E2: patients consuming minerals below the cut-off, I1: patients with positive PCR results for COVID-19, I2: patients with negative PCR results for COVID-19

As illustrated in Fig. 3, considering calcium, zinc, and iron, the number of patients in the E1 group (mineral consumption above the cut-off) is increasing, while there is a decline in the E2 group (mineral consumption below the cut-off). It shows that patients who consume less calcium, zinc, or iron than the cut-offs move to the COVID-19-positive group.

The risk of COVID-19 infection in the group with a daily calcium intake < 1138 mg per day was 5.2 times greater than in those who consume more than this value. Consumption of zinc < 9.7 mg per day was associated with a 1.5 times greater risk of COVID-19 infection. The risk of COVID-19 disease in the group with a daily Consumption of iron < 8.7mg per day was 2.1 times greater than those who consume more. On the other hand, consumption of iodine greater than 52.19 mg/day is associated with a greater risk of COVID-19 infection by three times.

## Discussion

This study sought to assess the association between specific dietary mineral intakes with COVID-19 infection in the older adult population. Taken together, dietary magnesium, iron, and potassium intake were significantly lower in COVID-19-positive compared to COVID-19-negative patients. Dietary intake of iron was significantly associated with the risk of COVID-19 infection. Patients hospitalized due to COVID-19 had a significantly lower intake of zinc. Dietary zinc intake was also significantly associated with the risk of hospitalization due to COVID-19 infection. In the dynamical system models, it was observed that dietary intakes of calcium, zinc, and iron below the specified cut-offs, as well as iodine intake above the cut-off, were associated with an elevated risk of COVID-19 incidence.

Based on our findings, there was a significant association between iron intake and COVID-19 incidence. Specifically, each unit increase in iron intake was linked to a 6% decrease in the risk of COVID-19 incidence. About 6.5% of human enzymes depend on iron, and virus replication is dependent on host cellular processes. The cellular iron status could affect viral pathogenesis and host response to viral infection [17, 18]. Besides, iron concentration is associated with macrophage function and cytokine production, influencing inflammatory responses to COVID-19 infection [19]. Iron deficiency also enhances impaired lung function and hypoxia, common traits of severe COVID-19 disease [20]. A recent meta-analysis showed that serum iron levels and hemoglobin indices are inversely associated with COVID-19 infection severity and mortality [21].

Our results indicate a significant association between dietary zinc intake and the risk of hospitalization due to COVID-19 infection. Specifically, each unit increase in zinc consumption was associated with approximately a 30% reduction in the risk of hospitalization. Zinc is an essential trace element for the immune system's growth and function [22]. Zinc deficiency due to inadequate intake or malabsorption results in immune system imbalance and increases the risk of inflammatory and infectious diseases [23]. An observational cohort study in 2021 demonstrated that lower serum zinc levels at admission are associated with more viral expansion, more unfavorable clinical presentation, longer time to become stable, and higher mortality [24]. Another prospective observational study also indicated that COVID-19 patients had significantly lower serum zinc levels, and zinc deficiency was associated with a higher rate of complications, prolonged hospitalization, and increased mortality [25].

Based on the results of the dynamical system, the risk of COVID-19 infection in the group with daily consumption of calcium < 1138.13 mg per day was 5.2 times greater than in those who consume more. According to previous studies, hypocalcemia was reported to be an independent risk factor for poor COVID-19 outcomes, together with C-reactive protein and interleukin 6. Hypocalcemia was also correlated with a more severe inflammatory response, hematologic complications, and mortality in COVID-19 patients [26–29]. Although previous studies showed that hypocalcemia is negatively associated with COVID-19, it should be considered that lower dietary intake of calcium is very difficult to cause hypocalcemia. An ecological study on 158 countries across the world demonstrated that the infection rate of COVID-19 increased by raising calcium consumption. It was justified that this may be due to the effect of higher calcium intakes on other chronic diseases like myocardial infarction [30].

Although there was no significant difference in dietary intake of iodine between COVID-19-positive and negative patients, the results of the dynamical system showed that the risk of COVID-19 infection in the group with daily consumption of iodine > 52.19 μg/day was associated with a greater risk of COVID-19 disease by three-fold. Iodine, a trace element required for thyroid function, is needed in small quantities to maintain health. According to previous studies, iodine has some roles in strengthening the immune system function and removing pathogens [31]. According to the Food and Nutrition Board of the Institute of Medicine, the recommended iodine intake is 150 µg/d. To achieve the iodine intake standards, the salt iodization program in Iran started in 1996; the main dietary source of iodine in our population is iodinated salt. In fact, in our population, iodine intake is correlated with salt intake. This harmful correlation may justify the adverse effect of iodine consumption on COVID-19 infection in our dynamical system analysis.

Numerous studies have examined changes in dietary intake during the COVID-19 pandemic. However, to our knowledge, there are only a few studies that have assessed the impact of dietary intakes on the risk of COVID-19 and its associated hospitalization. One of the strengths of this study was evaluating the association of past dietary intakes with the incidence of COVID-19 during its pandemic. Although COVID-19 is subsiding, our results may be helpful to prevent infectious disease crises in our population. We also applied advanced data analysis methods to determine the risk of COVID-19 disease in accordance with dietary intakes of minerals. Besides its strengths, some limitations should also be considered. First, to determine COVID-19 patients we only relied on PCR results from all clinical sites in Mashhad. Patients without a PCR positive result were considered as COVID-19 negative. It should be noted that this may prone the study to classification bias. Because some patients affected by Corona virus did not have a PCR test. Besides, we have not considered the frequency of COVID-19 infection and the time of disease occurrence in each person during the pandemic period. Individuals with dietary intake missing were excluded which may influence the generalizability of our results. It should also be considered that we did not define the system, therefore we did not mention the components, delays, or calibration methods. In this study, we only focused on the association of mineral intake with COVID-19 incidence and hospitalization. It should be noted that other factors, including chronic diseases, medications, taking supplements or dietary changes during the time may influence the association between mineral intake and risk of COVID-19 which have not been considered in this study.

## Conclusion

Higher dietary iron and zinc intake are associated with decreased risk of COVID-19 infection and hospitalization, respectively. Based on dynamical system results, higher intakes of calcium, zinc, and iron were associated with decreased susceptibility to COVID-19 infection.

## Data Availability

The datasets generated and/or analyzed during the current study are not publicly available due to university data ownership policies, but are available from the corresponding author on reasonable request.

## References

[1] WHO COVID-19 Dashboard. Geneva: World Health Organization, 2020. Available online: https://covid19.who.int/.

[2] The species Severe acute respiratory syndrome-related coronavirus (2020). classifying 2019-nCoV and naming it SARS-CoV-2. Nat Microbiol.

[3] van Doorn HR, Yu H. Viral respiratory infections. Hunter's tropical medicine and emerging infectious diseases: Elsevier; 2020. p. 284–8.

[4] Mehri A, Sotoodeh Ghorbani S, Farhadi-Babadi K, Rahimi E, Barati Z, Taherpour N, et al. Risk Factors Associated with Severity and Death from COVID-19 in Iran: A Systematic Review and Meta-Analysis Study. J Intensive Care Med. 2023:8850666231166344. 10.1177/08850666231166344 PMC10051011 36976873

[5] Calder PC, Carr AC, Gombart AF, Eggersdorfer M (2020). Optimal nutritional status for a well-functioning immune system is an important factor to protect against viral infections. Nutrients.

[6] Venter C, Eyerich S, Sarin T, Klatt KC (2020). Nutrition and the immune system: a complicated tango. Nutrients.

[7] Jothimani D, Kailasam E, Danielraj S, Nallathambi B, Ramachandran H, Sekar P (2020). COVID-19: poor outcomes in patients with zinc deficiency. Int J Infect Dis.

[8] Higgins JP (2002). Nonlinear systems in medicine. Yale J Biol Med.

[9] Banerjee S (2022). Dynamics of the COVID-19 pandemic: nonlinear approaches on the modelling, prediction and control. European Phys J Special Topics.

[10] Willy C, Neugebauer EAM, Gerngroß H (2003). The Concept of nonlinearity in complex systems. European J Trauma.

[11] Schoenenberger L, Schmid A, Tanase R, Beck M, Schwaninger M (2021). Structural analysis of system dynamics models. Simul Model Pract Theory.

[12] Ghayour-Mobarhan M, Moohebati M, Esmaily H, Ebrahimi M, Parizadeh SMR, Heidari-Bakavoli AR (2015). Mashhad stroke and heart atherosclerotic disorder (MASHAD) study: design, baseline characteristics and 10-year cardiovascular risk estimation. Int J Public Health.

[13] Hafezi SG, Seifi N, Bahari H, Mohammadi M, Ghasemabadi A, Ferns GA (2023). The association between macronutrient intakes and coronavirus disease 2019 (COVID-19) in an Iranian population: applying a dynamical system model. J Health Popul Nutr.

[14] Mahsa A, Zahra A, Hamid Heidarian M, Mehrangiz E-M, Majid G-M, Gorden F (2017). Validation of a short semi-quantitative food frequency questionnaire for adults: a pilot study. J Nutr Sci Dietetics.

[15] Ravasco P, Aranha MM, Borralho PM, da Moreira Silva IB, Correia L, Fernandes A (2010). Colorectal cancer: can nutrients modulate NF-kappaB and apoptosis?. Clin Nutr.

[16] Willett WC, Howe GR, Kushi LH (1997). Adjustment for total energy intake in epidemiologic studies. Am J Clin Nutr.

[17] Maggini S, Pierre A, Calder PC (2018). Immune function and micronutrient requirements change over the life course. Nutrients.

[18] Andreini C, Putignano V, Rosato A, Banci L (2018). The human iron-proteome. Metallomics.

[19] Cronin SJ, Woolf CJ, Weiss G, Penninger JM (2019). The role of iron regulation in immunometabolism and immune-related disease. Front Mol Biosci.

[20] Frise MC, Cheng H-Y, Nickol AH, Curtis MK, Pollard KA, Roberts DJ (2016). Clinical iron deficiency disturbs normal human responses to hypoxia. J Clin Investig.

[21] Zhou S, Li H, Li S (2022). The associations of iron related biomarkers with risk, clinical severity and mortality in SARS-CoV-2 patients: a meta-analysis. Nutrients.

[22] Read SA, Obeid S, Ahlenstiel C, Ahlenstiel G (2019). The role of zinc in antiviral immunity. Adv Nutr.

[23] Walker CF, Black RE (2004). Zinc and the risk for infectious disease. Annu Rev Nutr.

[24] Vogel-González M, Talló-Parra M, Herrera-Fernández V, Pérez-Vilaró G, Chillón M, Nogués X (2021). Low zinc levels at admission associates with poor clinical outcomes in SARS-CoV-2 infection. Nutrients.

[25] Jothimani D, Kailasam E, Danielraj S, Nallathambi B, Ramachandran H, Sekar P (2020). COVID-19: Poor outcomes in patients with zinc deficiency. Int J Infect Dis.

[26] Sun J-K, Zhang W-H, Zou L, Liu Y, Li J-J, Kan X-H (2020). Serum calcium as a biomarker of clinical severity and prognosis in patients with coronavirus disease 2019. Aging (Albany NY).

[27] Di Filippo L, Formenti AM, Rovere-Querini P, Carlucci M, Conte C, Ciceri F (2020). Hypocalcemia is highly prevalent and predicts hospitalization in patients with COVID-19. Endocrine.

[28] Wray JP, Bridwell RE, Schauer SG, Shackelford SA, Bebarta VS, Wright FL (2021). The diamond of death: Hypocalcemia in trauma and resuscitation. Am J Emerg Med.

[29] Bennouar S, Cherif AB, Kessira A, Bennouar D-E, Abdi S (2021). Vitamin D deficiency and low serum calcium as predictors of poor prognosis in patients with severe COVID-19. J Am Coll Nutr.

[30] Abdulah DM, Hassan A (2020). Relation of dietary factors with infection and mortality rates of COVID-19 across the world. J Nutr Health Aging.

[31] Bilal MY, Dambaeva S, Kwak-Kim J, Gilman-Sachs A, Beaman KD (2017). A role for iodide and thyroglobulin in modulating the function of human immune cells. Front Immunol.

